# Prenatal maternal mental health and resilience in the United Kingdom during the SARS-CoV-2 pandemic: a cross- national comparison

**DOI:** 10.3389/fpsyt.2024.1411761

**Published:** 2024-09-26

**Authors:** Swarali Datye, Marko Smiljanic, Rohan Shetti, Alison MacRae-Miller, Edwin van Teijlingen, Latha Vinayakarao, Eva M. J. Peters, Catherine Lebel, Lianne Tomfohr-Madsen, Gerald Giesbrecht, Minesh Khashu, Melanie L. Conrad

**Affiliations:** ^1^ Institute of Microbiology, Infectious Diseases and Immunology, Charité–Universitätsmedizin Berlin, Corporate Member of Freie Universität Berlin, Humboldt-Universität zu Berlin, Berlin Institute of Health, Berlin, Germany; ^2^ Neonatology and Pediatric Intensive Care, Faculty of Medicine, University of Augsburg, Augsburg, Germany; ^3^ Institute of Botany and Landscape Ecology, University of Greifswald, Greifswald, Germany; ^4^ Faculty of Environment, Julius von Payer Institute for Arctic and Subarctic Research, Jan Evangelista Purkyně University, Ústí nad Labem, Czechia; ^5^ Department of Family Medicine, University of British Columbia, Vancouver, BC, Canada; ^6^ Centre for Midwifery and Women’s Health, Bournemouth University, Bournemouth, United Kingdom; ^7^ University Hospitals Dorset NHS Foundation Trust, Poole, United Kingdom; ^8^ Psychoneuroimmunology Laboratory, Department of Psychosomatic Medicine and Psychotherapy, Justus-Liebig University, Gießen, Germany; ^9^ Department of Psychosomatic Medicine and Psychotherapy, Charité Universitätsmedizin Berlin, Berlin, Germany; ^10^ Department of Radiology, University of Calgary, Calgary, AB, Canada; ^11^ Alberta Children's Hospital Research Institute, University of Calgary, Calgary, AB, Canada; ^12^ Department of Pediatrics, University of Calgary, Calgary, AB, Canada; ^13^ Department of Psychology, University of Calgary, Calgary, AB, Canada; ^14^ Department of Community Health Sciences, University of Calgary, Calgary, AB, Canada; ^15^ Institute for Medical Psychology, Charité–Universitätsmedizin Berlin, Corporate Member of Freie Universität Berlin, Humboldt-Universität zu Berlin, Berlin Institute of Health, Berlin, Germany

**Keywords:** pregnancy, maternal mental health, depression, anxiety, resilience, social support, mixed methods

## Abstract

**Introduction:**

Prenatal mental health problems are associated with morbidity for the pregnant person, and their infants are at long-term risk for poor health outcomes. We aim to explore how the SARS-CoV-2 pandemic affected the mental health of pregnant people in the United Kingdom (UK), and to further identify resilience factors which may have contributed to varying mental health outcomes. We also aim to examine the quality of antenatal care provided during the pandemic in the UK and to identify potential inadequacies to enhance preparedness for future events.

**Methods:**

During June-November 2020, we recruited 3666 individuals in the UK for the EPPOCH pregnancy cohort (Maternal mental health during the COVID-19 pandemic: Effect of the Pandemic on Pregnancy Outcomes and Childhood Health). Participants were assessed for depression, anxiety, anger and pregnancy-related anxiety using validated scales. Additionally, physical activity, social support, individualized support and personal coping ability of the respondents were assessed as potential resilience factors.

**Results:**

Participants reported high levels of depression (57.05%), anxiety (58.04%) and anger (58.05%). Higher levels of social and individualized support and personal coping ability were associated with lower mental health challenges. Additionally, pregnant individuals in the UK experienced higher depression during the pandemic than that reported in Canada. Finally, qualitative analysis revealed that restrictions for partners and support persons during medical appointments as well as poor public health communication led to increased mental health adversities and hindered ability to make medical decisions.

**Discussion:**

This study revealed increased mental health challenges among pregnant individuals in the UK during the SARS-CoV-2 pandemic. These results highlight the need for reassessing the mental health support measures available to pregnant people in the UK, both during times of crisis and in general.

## Introduction

The COVID-19 pandemic was the most severe health crisis in modern times, with far-reaching consequences. Policymakers across the world resorted to unprecedented measures to contain the virus, including nationwide lockdowns, closing workplaces, schools and daycare centers, and broad restrictions on the public and social lives of citizens. Although these measures were strategies aimed at curbing the spread of the virus, they are widely recognized as having caused considerable psychosocial distress (1). Pregnant individuals may have been particularly susceptible to distress, as pregnancy is a vulnerable period in life marked by substantial physiological and psychological changes (2). The potential adverse outcomes of compromised perinatal mental health include miscarriage (3), preterm birth (4), low birth weight (4) and intrauterine growth restriction (5). In addition, maternal mental health problems during pregnancy can increase postnatal risks for infants such as delayed immune system development (6), respiratory infections, wheeze (7), allergies and asthma (8, 9) in early life.

Worldwide, several studies have observed an association between being pregnant or postpartum during the COVID-19 pandemic, and an increased risk of maternal mental illness (10–15). Research suggests that pregnant individuals living in the UK may have experienced more mental health difficulties in comparison with other developed countries. For instance, a multinational study by Ceulemans et al. (2021) conducted with pregnant and postpartum individuals in June and July 2020 reported living in the UK as a risk factor associated with higher levels of mental distress compared with other developed European nations (16). Filippetti et al. studied 150 expectant women living in the UK and found an increased prevalence of depression and anxiety related to the psychological impact of COVID-19 (17). Considering these findings in light of the conclusions of the MBRRACE-UK 2020 report, which showed that maternal suicide was the leading cause of maternal death within a year after pregnancy in the UK (18), it is evident that the aftermath of the mental health issues that arose among pregnant individuals during the pandemic is a significant public health concern.

Although reports have shown increased adverse mental health conditions in pregnant individuals in the UK, there is still a need for a large-scale, nationwide study examining the mental health of pregnant people during the pandemic. Further, a detailed assessment of the impact and mitigation of these intensified mental health issues among pregnant people is critical to improving mental health outcomes through better healthcare policies and practices in the future. The aim of this study was to investigate the impact of the COVID-19 pandemic on the extent of mental health adversity in a large pregnant population in the UK.

Mental health challenges during pregnancy encompass a range of emotional and psychological difficulties. Maternal depression and anxiety are prevalent during the prenatal period (19, 20) and can lead to adverse maternal and neonatal outcomes. Research has also shown that maternal anger is a significant emotional challenge during pregnancy and can potentially impact fetal development (21). Pregnancy-related anxiety, marked by specific fears related to childbirth, is also known to negatively affect the child’s physical growth in early life (22). These challenges, often intensified by the physiological changes of pregnancy, have important implications for both maternal and infant health.

Resilience is defined as an individual’s ability to cope with and recover from mental health problems or adversity, encompassing various protective factors such as physical activity, social and individualized support, and personal coping skills (23). Physical activity during the prenatal period is known to reduce symptoms of depression, contributing to improved mental health outcomes (24). Social and individualized support also plays a critical role in alleviating mental health issues during pregnancy (25). We included both, general social support and individualized support to capture their unique contributions to mental health given the challenging times of the COVID-19 pandemic. Social support, which includes support from a broader community, provides reassurance and a sense of belonging and has sustained health benefits to the mother and the child (26). Individualized support from partners or close family members offers critical emotional and practical assistance that can improve maternal mental health and is also known to positively influence fetal growth (27, 28). Additionally, improved personal resilience and coping skills are known to improve psychological wellbeing during pregnancy (29). In this study, we aim to identify specific resilience factors that were associated with improved mental health outcomes during the pandemic.

Early diagnosis of perinatal mental health challenges, followed by timely intervention could potentially deter progression into more serious mental illness, thereby improving quality of life for parents and their children. With this study, we also aim to assess participant perceptions of the quality of antenatal care received during the pandemic and the issues encountered in this regard. Additionally, we compared our results with the mental health findings reported in a comparable population in Canada during the pandemic to evaluate perinatal mental health outcomes between the two countries. The findings are of particular significance to clinicians and policymakers, offering a comprehensive analysis of the limiting factors relevant to perinatal mental health and maternal healthcare, particularly in stressful circumstances.

## Study design

This study presents a mixed methods analysis, where quantitative and qualitative data collected at a single time point (enrollment) are reported from the EPPOCH cohort. While the EPPOCH study was designed as a longitudinal cohort with multiple data collection points, only the baseline data collected at enrollment are analyzed and presented in this manuscript.

### Recruitment

From June to November 2020, we enrolled pregnant individuals in a cohort in the UK titled: Maternal mental health during the COVID-19 pandemic: Effect of the Pandemic on Pregnancy Outcomes and Childhood Health (EPPOCH). Institutional review board approval was obtained from Bournemouth University, Bournemouth, UK (ethics number: 32352) and Charité Universitätsmedizin Berlin (ethics number: EA2/086/22). Participants were recruited online, via advertisement over social media platforms such as Facebook and Twitter. Inclusion criteria for participation in the study were: ongoing pregnancy, residence in the UK, over 18 years of age, and the ability to read and write English. Study participation was voluntary and pregnant individuals self-enrolled for the study via a secure REDCap (30, 31) platform. Written informed consent was obtained from all individuals at the time of enrollment. The EPPOCH study is a sister cohort to the Pregnancy During the Pandemic (PdP) study in Canada, with both studies employing the same enrollment questionnaire (32). The PdP study recruited pregnant individuals across Canada via social media platforms. Inclusion criteria for the PdP study were: residence in Canada, ability to read and write English, and confirmed pregnancy <35 weeks of gestation. For comparison with our study, we used data from a subset of the PdP cohort, consisting of 1987 participants who filled out the questionnaire between 5- 20 April 2020 of the COVID-19 pandemic.

EPPOCH participants were assessed for eligibility before being admitted to the study, and 111 duplicates and invalid records with incorrect due dates were removed. The number of individuals who completed the entire enrolment questionnaire was 2826, while an additional 840 respondents completed a portion of the questionnaire (total n = 3666). We used listwise deletion to handle missing data, and for each analysis, only cases with complete data for all variables involved in that specific analysis were included. Sample sizes for each variable are reported in **Table 1**. Demographic information for the EPPOCH and PdP cohorts are given in **Supplementary Tables 1A, B** respectively.

**Table 1:** Descriptive statistics of the respondents including mental health measures, COVID-19 stressors and potential resilience factors.

Variable	Sample size (n)	Mean	Standard deviation	Median	Interquartile range	Min.	Max.
***Age (years)***	3503	31.45	5.20	31.42	7.17	19.08	46.75
Gestational age							
First trimester	558						
Second trimester	1756						
Third trimester	1960						
Cohabiting with a partner	3505 Yes 3126 No 379						
Covid-stressors (Higher values indicate higher mental health challenges)							
Distress at the perceived peak of the pandemic	3093	6.22	2.35	7	3	0	10
Distress in the week prior to enrollment	3093	4.51	2.64	5	5	0	10
Perceived decrease in the quality of care received	2957	58.77	29.31	62	41	0	100
Concern caused due to compromised antenatal care	2956	47.69	30.82	50	50	0	100
Loneliness during the COVID-19 pandemic	3055	70.87	25.54	75	36.5	0	100
Compromised financial situation and difficulties in paying for basic necessities due to COVID-19 pandemic	3158	25.08	27.06	15	50	0	100
Perceived risk to own life due to COVID-19	3145	45.59	25.93	50	41	0	100
Perceived risk of COVID-19 causing harm to the baby	3142	65.24	26.78	70	38	0	100
Mental health measures							
EPDS	3081	13.28	6.17	14	9	0	30
PROMIS anxiety (T-score)	3080	60.34	8.91	61.3	11.3	36.3	82.7
PRAQ	3067	23.06	5.92	22	8	10	40
PROMIS anger (T-score)	2836	56.16	9.59	56.7	12.4	32.9	83.3
Resilience factors							
GLTEQ	3666	55.97	26.17	55	40	0	119
ISEL	2897	37.96	7.46	39	11	12	48
SSEQ	2876	54.88	15.89	57	22	0	80
CD-RISC 2	2826	5.38	1.51	6	2	0	8

EPDS, Edinburgh Postnatal Depression Scale; PROMIS, Patient-Reported Outcomes Measurement Information System; PRAQ, Pregnancy-Related Anxiety Questionnaire; GLTEQ, Godin-Shephard Leisure-Time Exercise Questionnaire; ISEL, Interpersonal Support Evaluation List; SSEQ, Social Support Effectiveness Questionnaire; CD-RISC 2, Connor-Davidson Resilience Scale (2-item version).

### Demographics and general information

The participants provided information on their birth month and year, postcode, ethnic origin, and education. Data were also collected on marital status, parity, due date and whether the current pregnancy was planned. Clinical data such as preexisting maternal health conditions and COVID-19 infections during pregnancy were also recorded.

### Antenatal care during the pandemic

The participants’ perception of the effect of the COVID-19 pandemic on perinatal care was assessed using ‘Yes/No’ questions such as ‘Have you experienced changes in the way that perinatal care is delivered to you during the COVID-19 pandemic?’, ‘Have any of your antenatal care appointments been cancelled?’, ‘Do you currently have (or have you had) trouble accessing other health services during the COVID-19 pandemic?’ and ‘Are you able to bring your partner or support person to your appointments?`. Additionally, multiple choice questions (check boxes) were used to record changes made to birth plans and accessibility of specific health services. The complete set of questions used to assess changes to perinatal care is provided in **Supplementary Figure 1**.

### COVID-19 stressors

Participants were also asked to report their subjective distress level on a 10-point scale (0= no distress, 5= moderate distress, 10= extreme distress) at their perceived peak of the pandemic as well as in the week prior to enrollment. A score ≥ 4 was used to signify clinically concerning distress as per the standard of the Distress Thermometer validated scale (33). Participants were also asked questions centered on the impact of the pandemic on specific aspects of their lives and pregnancy, such as questions regarding household income, savings and difficulty in paying expenses. They also reported the degree to which they feared that their own lives or their babies’ lives were in danger due to COVID-19 as well as the level of their loneliness during the pandemic on a 100-point Likert scale ranging from 0 (Not at all) to 100 (Very much so). Finally, participants were asked the degree to which their relationships with friends and family outside of the household had been affected by the pandemic (0= it has strained our relationship, 50= not much has changed, 100= it has brought us closer together). The questions used to record COVID-19 stressors are provided in **Supplementary Figure 1**.

### Mental health assessment

#### Depression

Symptoms of depression were measured using the Edinburgh Postnatal Depression Scale (EPDS). The total scores on the EPDS range from 0-30, with higher scores indicating more severe depressive symptoms. Participants with scores ≥ 13 were defined as having clinically concerning symptoms of depression, as this threshold value has been reliably shown to have a positive predictive value (PPV) of 33%, specificity of 87% and sensitivity of 100% for identifying major symptoms of depression (34).

#### Generalized anxiety

Symptoms of generalized anxiety among the study population were evaluated using the Patient-Reported Outcomes Measurement Information System (PROMIS) Anxiety Adult 7-item short form (35). The items on this scale assess the frequency of anxious feelings experienced by respondents over the previous week on a scale of 1 (never) to 5 (always). The summed raw scores were converted to T-scores according to the standardized conversion table. T-scores between 60-69.9 indicated moderate anxiety, while scores ≥ 70 indicated severe anxiety symptoms.

#### Pregnancy-related anxiety

The 10-item Pregnancy-Related Anxiety Questionnaire (PRAQ) was used to assess symptoms of anxiety, focusing on the fear of birth, health of the baby and caring for the newborn (36). The total scores on this scale range from 10-40, with higher scores indicating greater levels of pregnancy-related anxiety. Since there is no established cut-off score for this scale, we used a median split to categorize lower and higher pregnancy-related anxiety symptoms, which reflects a similar use of this scale in previous studies (14, 37).

#### Anger

A 5-item version of the PROMIS Anger Short Form was used to evaluate feelings of annoyance and irritation experienced by the participants in the week prior to enrollment (35, 38). Respondents were asked how often they experienced feelings of anger on a 5-point scale: never (1), rarely (2), sometimes (3), often (4), and always (5). Raw totals were converted to T-scores. T-scores between 60 and 69.9 indicated moderate anger, while those ≥ 70 indicated severe symptoms of anger.

### Factors examined for their potential impact on mental health outcomes

#### Physical activity

Levels of physical activity in a typical week during the month prior to enrollment were documented using the Godin-Shephard Leisure-Time Exercise Questionnaire (GLTEQ) (39). Respondents with scores below 14 were interpreted as sedentary, those with scores between 14-23 were considered moderately active, while those with scores ≥24 were considered active. Moreover, participants were also asked whether their levels of physical activity had changed due to the COVID-19 pandemic, and responses were recorded on a 5-point scale with the following options: ‘Substantially increased’ (5), ‘Somewhat increased’ (4), ‘Somewhat decreased’ (3), ‘Substantially decreased’ (2), or ‘No change’ (1).

#### General social support

The general level of social support received by participants was assessed using the 12- item Interpersonal Support Evaluation List (ISEL) (40). Items in this scale seek information on various aspects of social support, including appraisal support (the perceived ability to have someone share one’s problems), belonging support (the perceived ability to have people to engage with), self-esteem support (the perceived ability to make a favorable comparison of oneself with others) and tangible support (perceived availability of material aid).

#### Support from a partner or support person

Individualized support received from a partner or another support person was assessed using the Social Support Effectiveness Questionnaire (SSEQ), which measures emotional, informational, and task support received by respondents (41). Higher scores indicate better support on both support scales.

#### Personal coping ability

The Connor-Davidson Resilience Scale (CD-RISC 2) was used to gauge participants’ perceptions of their own ability to deal with stressful events (42). The scale comprises 2 items assessing the ability to adapt to changing life situations and to recover after experiencing adversity. Total scores range from 0 to 8, with higher scores indicating better coping ability.

### Data analysis

#### Quantitative analyses

Questionnaire data were examined manually for precision and reliability prior to statistical analysis. Quantitative analyses were performed using R (version 4.0.4) (43). Descriptive statistics were used to summarize the main research variables. Associations between maternal mental health measures and resilience factors were reported using Pearson’s bivariate correlations. Multivariate binary logistic regression models were used to identify predictor variables associated with mental health outcomes. The main predictors of interest were resilience factors, including physical activity (Godin-Shephard Questionnaire), social and individualized support (ISEL, SSEQ respectively) and personal coping skills (CD-RISC 2). Covariates included cohabiting with a partner, planned pregnancy, maternal age, parity, and presence of physical conditions before pregnancy. We included representative variables of depression, generalized anxiety, and anger in our regression analyses as mental health outcome variables. The binary classification of mental health variables was carried out on the basis of previously defined threshold scores suggested in the literature (EPDS score ≥13, PROMIS T-score for anxiety and anger ≥60) (34, 35).

The Caret package (44) was used to train and evaluate the models. The dataset was first randomly divided into training (80%) and testing (20%) sets. Several classification model families were fitted and evaluated with a 5-fold cross-validation procedure used to estimate the independent performance of the models and prevent overfitting. The final chosen model of multivariate logistic regression was fitted on the entire training dataset and evaluated using the initial testing set with the Receiver Operating Characteristic (ROC) curve. Additionally, accuracy, sensitivity, specificity, and kappa scores for the 0.5 probability threshold were reported to provide an overall evaluation of the model’s performance. Finally, odds ratios for each predictor variable of the multivariate logistic models were reported with their 95% confidence interval (CI). For a cross-national comparison of the effect of resilience factors on mental health measures, Pearson’s bivariate correlations of mental health measures with resilience factors in the EPPOCH and the Canadian PdP cohorts were compared using Fisher Z-Transformation.

#### Qualitative analyses

To gain insight into the specific challenges encountered by pregnant individuals in the UK during the pandemic, qualitative analysis was performed on participant responses (n=1169) to the open question: “Do you have any other comments regarding the changes to antenatal care or the involvement of your partner or your support person?” This analysis adhered to the thematic analysis guideline described by Braun and Clarke (2006) (45). First, two independent researchers familiarized themselves with the dataset by reading through the participant responses. The text responses were then imported into the NVivo 14 software (Lumivero), and autocoded for common words and phrases. Nodes for significant concepts, phrases, or ideas (that were categorized by the software), were then reviewed to identify recurring patterns and themes. Themes were determined based on relevance to antenatal care during the pandemic, node frequency and the intensity of expressed emotions (positive/negative). From this analysis, the two most common themes were identified and named as follows: 1) Restrictions for partners and support persons 2) Poor public health communication. The interpretation of themes was conducted in the context of existing literature on antenatal care during the pandemic. In addition to this, illustrative quotes were selected that exemplified each theme to ensure that participant voices were accurately represented.

## Results

### Demographics and general information

The mean age of the participants was 31.42 ± 5.2 years. Most of the participants (89.19%) were married or living with a partner. Of the study population, 15.92% had no other children, 54.75% had one child, 17.89% had two other children, and 11.44% had three or more other children. The percentage of participants holding a bachelor’s degree was 35.98%, followed by those who completed trade/technical/vocational school or business/community college (29.81%), those with graduate degrees (17.57%) and those who completed high school education (15.15%). Only 1.49% did not have a high-school diploma. Regarding COVID-19 infection status, the majority of participants (89.80%) reported no infections, while 1.40% confirmed diagnosis with COVID-19, and 8.80% suspected infection.

### Antenatal care during the pandemic

Among the 3130 participants, 94.50% reported a change in the way perinatal care was delivered during the pandemic, and 94.35% said that their partner or support person was not allowed to accompany them to perinatal care appointments. Changes to birth plans due to the pandemic (n=3123) were reported by 19.30% of individuals, out of whom 6.86% changed their planned birth location, 12.80% changed their intended support persons, while 5.27% made changes to their childcare arrangements. Additionally, 46.13% of the population had trouble accessing allied healthcare services during the pandemic, including massage (15.59%), physiotherapy (12.87%), chiropractic and acupuncture (6.81%) and psychological support services (6.13%). On a scale of 0-100, the mean scores for a perceived decrease in the quality of care and the concern felt by participants due to compromised care were 58.77 ± 29.31 and 47.69 ± 30.82, respectively (**Table 1**).

### COVID-19 stressors

On the distress thermometer, 85.35% of participants reported clinically concerning distress symptoms at their perceived peak of the pandemic, while 62.46% reported experiencing clinically concerning distress in the week prior to completing the survey. Additionally, the mean, median, and range of scores for common pandemic-induced stressors are shown in **Table 1**. On a scale of 0-100, with increasing scores implying higher COVID-19 related stress, the mean score indicating a compromised financial situation and difficulty in affording basic needs during the pandemic was 25.08 ± 27.06. The mean scores for perceived risk to the participant’s own life and to the baby’s life were 45.59 ± 25.93 and 65.24 ± 26.78, respectively. The mean score on the same scale for loneliness experienced during the pandemic was 70.87 ± 25.54 (**Table 1**).

### Mental health assessment

On the depression questionnaire, 57.05% of the participants showed clinically concerning symptoms. On the generalized anxiety questionnaire, 44.93% of respondents reported moderate anxiety, whereas 13.11% reported severe symptoms of anxiety. On the pregnancy-related anxiety questionnaire, the mean score was 23.06 ± 5.92. On the anger questionnaire, 58.05% of the participants reported moderate to severe anger in the week prior to their enrollment. Measures of central tendency are presented in **Table 1**. Depression scores were strongly correlated with anxiety (r = 0.85, 95% CI: 0.83 – 0.86, p<0.0001) and anger scores (r = 0.64, 95% CI: 0.62 – 0.66, p<0.0001), while all three of these scores were moderately to weakly correlated with pregnancy-related anxiety scores (**Table 2**).

**Table 2:** Pearson’s bivariate correlations among mental health measures and resilience factors (95% confidence interval in parentheses).

	EPDS	PROMIS anxiety	PRAQ	PROMIS anger	GLTEQ	ISEL	SSEQ
**EPDS**	**1 (1 - 1)**	**—**	**—**	**—**	**—**	**—**	**—**
**PROMIS anxiety**	**0.85 (0.83 - 0.86)**	**1 (1 - 1)**	**—**	**—**	**—**	**—**	**—**
**PRAQ**	**0.49 (0.46 - 0.51)**	**0.5 (0.48 - 0.53)**	**1 (1 - 1)**	**—**	**—**	**—**	**—**
**PROMIS anger**	**0.64 (0.62 - 0.66)**	**0.58 (0.56 - 0.61)**	**0.34 (0.31 - 0.38)**	**1 (1 - 1)**	**—**	**—**	**—**
**GLTEQ**	-0.01(-0.04 - 0.03)	0.00 (-0.03 - 0.04)	-0.03 (-0.07 - 0.01)	0.00 (-0.03 - 0.04)	**1 (1 - 1)**	**—**	**—**
**ISEL**	**-0.40 (-0.44 - -0.38)**	**-0.33 (-0.37 - -0.31)**	**-0.24 (-0.28 - -0.21)**	**-0.36 (-0.39 - -0.32)**	0.05 (0.01 - 0.08)	**1 (1 - 1)**	**—**
**SSEQ**	**-0.37 (-0.41 - -0.35)**	**-0.30 (-0.35 - -0.28)**	**-0.20 (-0.24 - -0.17)**	**-0.44 (-0.47 - -0.41)**	0.01 (-0.03 - 0.04)	**0.5 (0.47 - 0.53)**	**1 (1 - 1)**
**CD-RISC 2**	**-0.42 (-0.45 - -0.39)**	**-0.38 (-0.41 - -0.35)**	**-0.28 (-0.31 - -0.24)**	**-0.32 (-0.35 - -0.28)**	**0.11 (0.08 - 0.15)**	**0.32 (0.29 - 0.35)**	**0.2 (0.17 - 0.24)**

Significant results (p <0.01) are shown in bold. Only those records for which data on all the above measures was obtained were considered for this analysis.

EPDS, Edinburgh Postnatal Depression Scale; PROMIS, Patient-Reported Outcomes Measurement Information System; PRAQ, Pregnancy-Related Anxiety Questionnaire; GLTEQ, Godin-Shephard Leisure-Time Exercise Questionnaire; ISEL, Interpersonal Support Evaluation List; SSEQ, Social Support Effectiveness Questionnaire; CD-RISC 2, Connor-Davidson Resilience Scale (2-item version).

### Potential resilience factors

#### Physical activity

The mean weekly leisure score on the physical activity Questionnaire was 55.97 ± 26.17, demonstrating that the population was active (**Table 1**). Of the participants, 83.86% reported a significant change in their physical activity levels due to the pandemic, of which 86.22% reported decreased physical activity, while 13.77% reported an increase. However, we did not find a significant correlation between physical activity questionnaire scores and mental health measures.

#### Social and individualized support and personal coping ability

As social and individualized support and personal coping ability are known to have a positive impact on mental health (40–42), we assessed these factors using the ISEL, SSEQ, and CD-RISC-2 scales, respectively. The mean score for general social support was 37.96 ± 7.46, while the mean score for individualized support was 54.88 ± 15.89 which are consistent with previous reports in pregnant people (14). The mean scores of personal coping were 5.38 ± 1.51, as shown in **Table 1**. Both, social and individualized support as well as personal coping scores were negatively correlated with all mental health measures including depression, anxiety, pregnancy-related anxiety and anger. (**Table 2**).

### Factors in association with depression, anxiety, and anger symptoms

Binary logistic regression was used to determine which factors were associated with depression, anxiety, and anger in the EPPOCH cohort. As outlined in **Table 3**, high levels of social and individualized support and coping ability were associated with lower levels of depression, anxiety and anger symptoms. Additionally, an increase in maternal age was associated with lower odds of anxiety and anger symptoms. Finally, pre-existing physical health conditions before pregnancy were associated with higher depression scores. The ROC curves along with the accuracy, sensitivity, specificity, and kappa scores are included in **Supplementary Figure 2**.

**Table 3:** Multivariate model predicting mental health outcomes (EPDS, PROMIS anxiety and PROMIS anger) in response to resilience factors (physical activity (GLTEQ), social support (ISEL), individualized support (SSEQ), personal coping skills (CD-RISC 2)) with covariates (maternal age, parity, cohabiting with partner, planned pregnancy and presence of physical conditions before pregnancy).

Variable	Beta coefficient	Standard error	Z value	p value	Odds ratio	95% CI	
						Lower	Upper
EPDS							
Intercept	0.163	0.067	2.434	0.015	1.177	1.032	1.342
Maternal age	-0.180	0.073	-2.469	0.013	0.835	0.724	0.963
Parity	0.108	0.072	1.489	0.136	1.114	0.966	1.284
Cohabiting with partner	0.022	0.075	0.298	0.765	1.022	0.883	1.184
Planned pregnancy	-0.123	0.071	-1.736	0.082	0.884	0.769	1.016
**Physical conditions before pregnancy**	**0.183**	**0.067**	**2.725**	**0.006**	**1.200**	**1.053**	**1.369**
GLTEQ	0.159	0.069	2.309	0.021	1.172	1.024	1.342
**ISEL**	**-0.310**	**0.084**	**-3.704**	**<0.001**	**0.732**	**0.621**	**0.864**
**SSEQ**	**-0.528**	**0.080**	**-6.580**	**<0.001**	**0.589**	**0.504**	**0.690**
**CD- RISC 2**	**-0.608**	**0.076**	**-7.968**	**<0.001**	**0.544**	**0.469**	**0.632**
PROMIS anxiety							
Intercept	0.063	0.065	0.977	0.328	1.065	0.938	1.209
**Maternal age**	**-0.260**	**0.071**	**-3.667**	**<0.001**	**0.770**	**0.670**	**0.886**
Parity	0.092	0.071	1.297	0.195	1.096	0.954	1.260
Cohabiting with partner	-0.106	0.072	-1.475	0.140	0.899	0.781	1.035
Planned pregnancy	-0.050	0.069	-0.731	0.465	0.950	0.830	1.089
Physical conditions before pregnancy	0.114	0.065	1.759	0.078	1.121	0.987	1.274
GLTEQ	0.128	0.066	1.921	0.054	1.136	0.997	1.295
**ISEL**	**-0.321**	**0.078**	**-4.080**	**<0.001**	**0.725**	**0.621**	**0.846**
**SSEQ**	**-0.359**	**0.074**	**-4.820**	**<0.001**	**0.698**	**0.603**	**0.808**
**CD- RISC 2**	**-0.490**	**0.072**	**-6.807**	**<0.001**	**0.612**	**0.532**	**0.705**
PROMIS Anger							
Intercept	-0.591	0.069	-8.466	<0.001	0.554	0.483	0.635
**Maternal age**	**-0.197**	**0.075**	**-2.615**	**0.008**	**0.821**	**0.709**	**0.952**
Parity	0.148	0.073	2.021	0.043	1.159	1.004	1.339
Cohabiting with partner	-0.024	0.073	-0.335	0.737	0.975	0.845	1.126
Planned pregnancy	0.072	0.072	0.998	0.318	1.075	0.933	1.239
Physical conditions before pregnancy	0.126	0.069	1.818	0.069	1.134	0.990	1.299
GLTEQ	0.133	0.070	1.895	0.058	1.143	0.995	1.312
**ISEL**	**-0.333**	**0.080**	**-4.168**	**<0.001**	**0.716**	**0.612**	**0.838**
**SSEQ**	**-0.671**	**0.079**	**-8.456**	**<0.001**	**0.511**	**0.438**	**0.597**
**CD- RISC 2**	**-0.495**	**0.075**	**-6.563**	**<0.001**	**0.609**	**0.525**	**0.706**

Significant results (p < 0.01) are shown in bold.

EPDS, Edinburgh Postnatal Depression Scale; PROMIS, Patient-Reported Outcomes Measurement Information System; GLTEQ, Godin-Shephard Leisure-Time Exercise Questionnaire; ISEL, Interpersonal Support Evaluation List; SSEQ, Social Support Effectiveness Questionnaire; CD-RISC 2, Connor-Davidson Resilience Scale (2-item version).

### Comparison of mental health and resilience in the EPPOCH and PdP pandemic pregnancy cohorts

Additionally, we performed comparisons of mental health and resilience between the UK and Canada using the EPPOCH and PdP cohort data. Both pregnancy cohorts were established during the pandemic, using the same enrollment questionnaire. The EPPOCH-UK and PdP cohorts show comparable demographic characteristics in terms of age and ethnicity. The mean age of participants was similar, with EPPOCH-UK at 31.45 ± 5.20 years and PdP at 32.4 ± 4.2 years. Regarding ethnicity, both cohorts had a predominantly Caucasian composition, with 89.58% of EPPOCH-UK participants identifying as English/Welsh/Scottish/Northern Irish/Irish, and 87.10% of PdP participants identifying as Caucasian. With regards to COVID-19 infections, both the EPPOCH and PdP cohorts reported low rates of confirmed COVID-19 cases. In EPPOCH, 1.40% of participants had confirmed COVID-19 diagnoses, while 8.80% reported suspected infections. The PdP study reported only one confirmed case and 25 suspected cases of COVID-19 in the study population.

**Figure 1:**
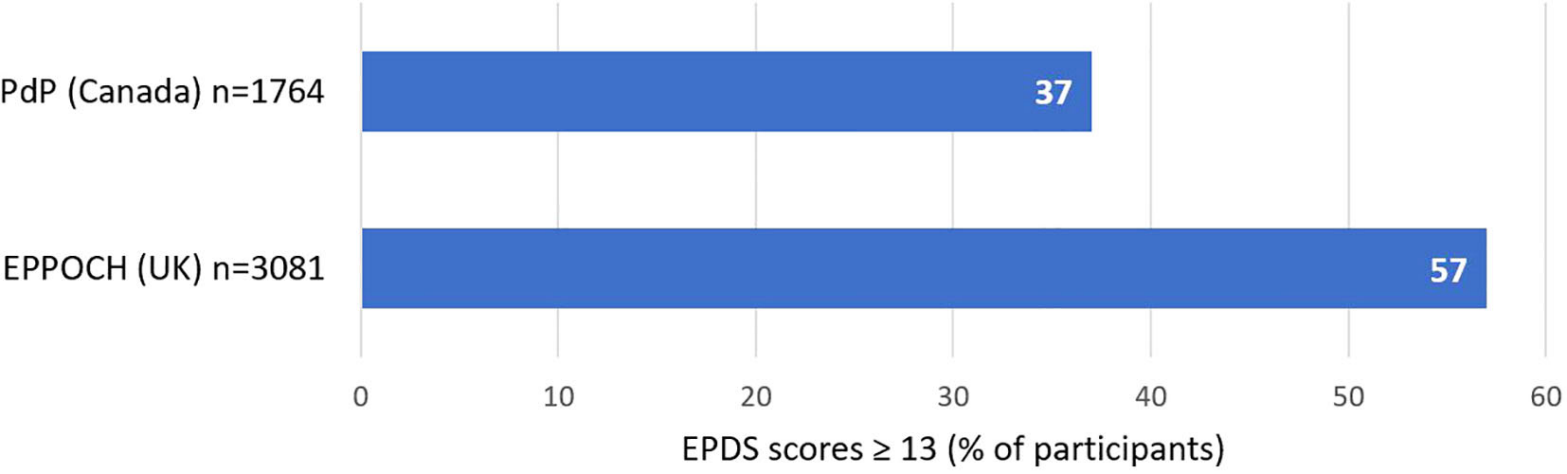
A comparison of clinically concerning depression among pregnant populations in the EPPOCH (UK) and PdP (Canada) cohorts during the pandemic in 2020, assessed by the EPDS questionnaire.

Regarding mental health measures, EPPOCH data showed that levels of clinically concerning depression were 54% higher in the UK than the PdP study in Canada (**Figure 1**). Generalized anxiety scores were similar across both cohorts, with 56.60% of PdP participants (n=1757) and 58.04% of EPPOCH-UK participants (n=3080) reporting clinically elevated anxiety symptoms. Pearson’s bivariate correlations indicated that the associations between social and individualized support, personal coping skills, and improved mental health outcomes were mirrored in both cohorts, while correlations between physical activity and mental health differed significantly, as shown in **Table 4**.

**Table 4:** Comparison of Pearson’s bivariate correlations of resilience factors with mental health measures between EPPOCH-UK and PDP cohorts.

	EPPOCH - UK	PdP - Canada		
	r_1_	r_2_	z value	*p value*
**GLTEQ~EPDS**	**-0.01**	**-0.10**	**3.21**	**< 0.01**
**GLTEQ~PROMIS anxiety**	**0.00**	**-0.10**	**3.57**	**< 0.01**
**GLTEQ~PRAQ**	**-0.03**	**-0.14**	**3.95**	**< 0.01**
**GLTEQ~PROMIS anger**	**0.00**	**-0.11**	**3.93**	**< 0.01**
ISEL~EPDS	-0.40	-0.35	-1.89	0.06
ISEL~PROMIS anxiety	-0.33	-0.26	-2.50	0.01
ISEL~PRAQ	-0.24	-0.24	0.00	1.00
ISEL~PROMIS anger	-0.36	-0.32	-1.47	0.14
SSEQ~EPDS	-0.37	-0.38	0.38	0.70
SSEQ~PROMIS anxiety	-0.30	-0.31	0.36	0.72
SSEQ~PRAQ	-0.20	-0.20	0.00	1.00
SSEQ~PROMIS anger	-0.44	-0.44	0.00	1.00
CD-RISC 2~EPDS	-0.42	-0.42	0.00	1.00
CD-RISC 2~PROMIS anxiety	-0.38	-0.35	-1.11	0.27
CD-RISC 2~PRAQ	-0.28	-0.26	-0.69	0.48
CD-RISC 2~PROMIS anger	-0.32	-0.30	-0.71	0.48

Significant results, indicating differences between correlations in both cohorts (p < 0.01), are shown in bold.

EPDS, Edinburgh Postnatal Depression Scale; PROMIS, Patient-Reported Outcomes Measurement Information System; PRAQ, Pregnancy-Related Anxiety Questionnaire; GLTEQ, Godin-Shephard Leisure-Time Exercise Questionnaire; ISEL, Interpersonal Support Evaluation List; SSEQ, Social Support Effectiveness Questionnaire; CD-RISC 2, Connor-Davidson Resilience Scale (2-item version).

### Qualitative analysis of antenatal care during the pandemic

Qualitative analysis conducted on responses to the free-text question, “Do you have any other comments regarding the changes to antenatal care or the involvement of your partner or your support person?” (n=1169) identified two major themes regarding antenatal care in the UK: 1) Restrictions for partners and support persons, and 2) Poor public health communication.

Theme 1 – Restrictions for partners and support persons.

The most prominent recurrent theme and negative aspect of antenatal care during the pandemic, as highlighted by our study participants, pertains to the restriction of partners or support persons attending scans and appointments. Of 3178 respondents, only 180 (5.66%) were able to bring a support person with them and this was especially stressful for first time and complicated pregnancies.

*“It has been very frustrating for us to not have my partner at appointments for what is a complicated pregnancy with significant decision-making required on our part in terms of mode of delivery. It will be my partner’s first and only child, so very saddening and isolating for him to miss out on antenatal information and bonding opportunities.”*

Respondents further remarked on the perceived unfairness of the differing rules regarding partner attendance for private versus public healthcare scans. Pregnant individuals expressed that if they paid out-of-pocket for a private ultrasound, they were allowed to have a partner present. However, this allowance did not extend to NHS-provided scans, which they were required to attend alone. This was viewed as inequitable, as respondents felt they should not have to pay for private care in order to have their partner accompany them.

*“My partner can attend a private scan but not an NHS scan, for this reason I have paid to go to a 16-week private scan. It does not make sense and is very unfair to those in financial poverty.”*

Restrictions for partners and support persons reached a critical juncture during the reopening of public establishments after a lockdown. Despite the reopening of various venues, such as pubs, shopping malls and swimming pools, many respondents expressed anger that they were still not allowed to bring a support person to their antenatal appointments. This discrepancy in public health guidelines was perceived as highly problematic by pregnant participants.

*“I think it is a disgrace that partners can’t come to scans. It affects me and my partner’s mental wellbeing and considering people can go shopping and even go to pubs but fathers to be can’t go to the antenatal appointments is such a failure in antenatal care, especially nowadays where we know how important paternal attachment is and therefore fathers should be included in the care of their baby.”*

Theme 2 – Poor public health communication.

In addition to restrictions for partners and support persons, a high proportion of our EPPOCH study participants voiced issues regarding poor communication from public health authorities. Respondents also expressed anxiety and frustration regarding the minimal contact with their doctors and midwives. Concerns included insufficient updates, unclear guidance and difficulty obtaining information and answers to their questions.

*“I feel unsure about my birthing plan, next appointments, I feel there is a lack of communication between myself and the midwives and I’m 33 weeks pregnant nearly and don’t even know who is supporting me.”*

“*I have had no antenatal appointments and have been told the only thing I can do is read online, on the NHS website for information.*”

Pregnant individuals also described instances where the information they received from public health authorities was ambiguous, contradictory, or difficult to interpret. This led to uncertainty regarding public health directives and recommendations. Compounding these issues, as individual NHS trusts were allowed to establish their own rules and protocols, participants noted the inconsistency of public health decisions, which lead to misunderstandings and loss of trust in the public health authorities.

*“My partner wasn’t allowed to anything, until the day they announced that he could attend the 20-week scan. However, three hospital workers said he wasn’t allowed to attend. We had to show them the news from NHS/government for them to let us through. Only the scanning person knew he was allowed in. If it wasn’t for our own knowledge, he would be sent away.”*

*“Seems inconsistent between NHS trusts. Calls into question the value of these appointments when different decisions are being made across the country.”*

These communication breakdowns, characterized by both insufficient and unclear messaging, emerged as significant barriers that impacted the ability of pregnant individuals to effectively navigate their medical decisions during the pandemic.

## Discussion

The data for the EPPOCH study was gathered between June 2020 and November 2020, which was a period marked initially by the easing of restrictions imposed after the first wave of the pandemic in the UK, followed by further lockdowns in the wake of the second wave of COVID-19 infections. We demonstrated that pregnant individuals in the UK experienced substantial mental health adversity during the pandemic in terms of depression, anxiety and anger.

To determine the extent of maternal mental health adversity experienced by our EPPOCH study participants during the pandemic, we referred to depression data from published literature on pre-pandemic pregnancy cohorts in the UK. The Avon Longitudinal Study of Parents and Children (ALSPAC), which monitored two generations of pregnant people for depression using the EPDS questionnaire, reported clinically concerning depression in 17% of the first generation (1990-1992) and 25% of the second generation (2012-2016) (EPDS scores ≥ 13) (46). The EPPOCH data, collected in the UK during the pandemic showed substantially increased levels of clinically concerning depression on the EPDS (2.28 times higher) than the 2012-2016 time point in the ALSPAC cohort. Additionally, a study assessing depression in pregnant individuals in London between 2014-2016 reported a mean EPDS score of 7.9 and a median score of 7.0 (47). In comparison with this pre-pandemic data, the depression scores from the EPPOCH study show that the COVID-19 pandemic was associated with substantially higher levels of maternal depression in the UK.

In a 2011-2012 multinational assessment of EPDS scores between pregnant people in the UK and ten other European countries, Lupattelli et al. showed a higher prevalence of severe perinatal depressive symptoms in the UK (EPDS score ≥ 22) (48). This indicates a preexisting trend towards decreased maternal mental health and calls for further studies to examine the causal factors for increased levels of depression in the UK. With respect to depression during the pandemic, EPDS scores from our EPPOCH cohort identified the UK as having substantially higher levels of perinatal depression during the pandemic, when compared to other European populations during the same time period (Switzerland, the Netherlands, Norway and Ireland) (16). Though our evidence demonstrates increased depression scores in the UK compared to other countries, we acknowledge that there could be several factors potentially responsible for this disparity, including infection rates, government mandates, immigration policies, employment conditions, and the demographics of participants in the UK and other European countries; a topic that would be beneficial to examine in future studies.

We conducted a cross-national comparison of our EPPOCH (UK) data and that of our sister cohort, the PdP (Canada) study (32). These cohorts are uniquely comparable in that the EPPOCH and PdP studies used the same enrollment questionnaire and recruited pregnant people during the pandemic with similar demographics. We found that maternal depression scores were substantially higher in the EPPOCH cohort in comparison with Canada. The low COVID-19 infection rates in both cohorts coupled with a high prevalence of mental health challenges, suggest that the observed psychological impacts may be more strongly associated with pandemic-related restrictions and societal disruptions rather than direct viral infections. Moreover, both cohorts demonstrated improved mental health outcomes in response to increased social and individualized support, as well as improved personal coping abilities. These findings align with research on perinatal mental health and social support in Canada (14). This also suggests that strategies aimed towards enhancing social support networks and developing personalized coping mechanisms may be effective in mitigating the mental health challenges in both countries. The differences in the correlations between physical activity and mental health outcomes across UK and Canadian cohorts may be attributed to variations in pandemic-related restrictions between the two countries, such as differences in lockdown measures and closures of recreational facilities, as well as other societal conditions such as access to outdoor spaces (49).

During the EPPOCH study recruitment period from June to November 2020, the UK experienced fluctuating COVID-19 rates. At the beginning of this period, the number of new cases was relatively high, which then decreased in early July. However, there was a notable increase in cases and hospitalizations towards the end of the recruitment period (50). Hence, various factors such as compromised healthcare, lack of support networks, economic impacts, social isolation and hospitalization/deaths among family or friends may have played a role in exacerbating mental health problems during this period. A detailed investigation of the causes of the intensified mental health challenges observed in pregnant individuals in the UK warrants further research.

A majority of our EPPOCH study participants reported inadequate antenatal care during the pandemic, which is in agreement with several other studies (51–53). Qualitative analysis of free-text responses, identified two major themes that negatively affected antenatal care in the UK during the pandemic. The first prominent theme, “Restrictions for partners and support persons,” revealed significant frustration among respondents over the inability to have partners or support persons attend medical appointments and scans. These restrictions were part of broader infection control measures implemented nationally to minimize virus transmission, rather than being based on individual COVID-19 status. A UK study reported that among pregnant mothers who had an ultrasound during the pandemic (n = 565), 65.5% reported being alone during the examination, resulting in significantly increased anxiety for both parents (54). The impact of these restrictions was further exacerbated by the perceived inequity, as private ultrasound providers often allowed partner attendance while NHS-based scans did not, highlighting disparities in access to supportive care during pregnancy (54).

Additionally, as public venues such as restaurants reopened, participants felt that these restrictions were unfairly maintained. For the second major theme “Poor public health communication” respondents addressed the anxiety caused by non-communication and/or contradictory messaging from public health authorities and healthcare providers. Respondents reported a lack of updates, unclear guidance, and difficulty obtaining information – all of which hindered their ability to navigate medical decisions during the pandemic. This aligns with findings from other studies in the UK that reported rapidly changing guidance and unclear public health messaging related to pregnancy and birth, with limited access to information about COVID-19 restrictions lead to increased fear and anxiety among expectant mothers during the pandemic (53, 55).

Moreover, our findings indicate that pregnant individuals with higher levels of social support, individualized support, and personal coping ability demonstrated fewer symptoms of depression, anxiety, and anger. This suggests that the presence of support networks and coping skills helped mitigate mental health challenges during the COVID-19 pandemic in the UK, despite disruptions to medical care. These results align with previous research emphasizing the protective role of social support in perinatal mental health (25, 56). Our findings suggest that targeted interventions enhancing support networks and coping abilities may be particularly beneficial for reducing perinatal mental health problems. Such interventions could include developing community-based initiatives to foster social support networks and introducing resilience-building programs focused on stress management and emotional regulation in prenatal care settings. These are key considerations for policymakers seeking to improve healthcare for pregnant individuals in the UK, both in routine circumstances as well as in crisis situations. Our findings also emphasize the need for policymakers to carefully consider the impact of visitation restrictions on maternal mental health and the implementation of flexible, evidence-based visitation policies that balance infection control with the emotional and practical support needs of pregnant individuals and their families. Furthermore, prioritizing clear, consistent, and timely public health communication is crucial. Policymakers should establish robust communication channels to ensure that pregnant individuals receive accurate, up-to-date information about care provisions and safety measures. Additionally, we suggest establishing a multidisciplinary committee including healthcare professionals, public health experts, and patient representatives, as a strategy to maintain continuity of care during crises and to develop emergency preparedness plans for healthcare during public health crises.

The study makes a significant contribution to the existing literature by providing a comprehensive analysis of prenatal mental health in the UK during the SARS-CoV-2 pandemic. It highlights the increased mental health challenges faced by pregnant individuals in the UK compared to other developed countries, using a large-scale, nationwide dataset. The research identifies specific UK-related factors, such as the impact of partner restrictions during medical appointments and poor public health communication, that exacerbated mental health adversities. Additionally, the study offers valuable insights into resilience factors that can mitigate mental health problems. These findings are crucial for informing healthcare policies and practices to better support pregnant individuals during crises and beyond.

### Study limitations

Pregnant individuals experiencing higher levels of mental health issues as a result of the COVID-19 pandemic may have been more likely to participate in pandemic-related mental health studies, leading to selection bias.

## Data Availability

The raw data supporting the conclusions of this article will be made available by the authors, without undue reservation.
