# Supplementary material for: Prenatal maternal mental health and resilience in the United Kingdom during the SARS-CoV-2 pandemic: a cross- national comparison
Source: Front Psychiatry. 2024 Sep 26;15:1411761. doi: 10.3389/fpsyt.2024.1411761 (PMC11466367; doi:10.3389/fpsyt.2024.1411761)
Supplement: Supplementary file 1 [file Image1.pdf]

EPPOCH- COVID stressors and perinatal care during the pandemic

Has your monthly household income changed because of the COVID-19 pandemic?

- ◯ Substantially decreased
- ◯ Somewhat decreased
- ◯ No change
- ◯ Somewhat increased
- ◯ Substantially increased

Have your household savings changed because of the COVID-19 pandemic?

- ◯ Substantially decreased
- ◯ Somewhat decreased
- ◯ No change
- ◯ Somewhat increased
- ◯ Substantially increased

Have changes in your personal financial situation during the COVID-19 pandemic made it if hard for you to pay for the basics like food, housing, medicine, and/or heating?

Not hard at all — Somewhat hard — Very hard

(Place a mark on the scale above)

How much do (did) you think your life is (was) in danger during the COVID-19 pandemic?

Not at all — Somewhat — Very much

(Place a mark on the scale above)

How much do (did) you think your baby's life is (was) in danger at any time during the COVID-19 pandemic?

Not at all — Somewhat — Very much

(Place a mark on the scale above)

How much are you worried that exposure to the COVID-19 virus will harm your baby?

Not at all — Somewhat — Very much

(Place a mark on the scale above)

Have you experienced changes in the way that antenatal care is delivered to you during the COVID-19 pandemic?

- ◯ Yes
- ◯ No

Have any of your antenatal care appointments been cancelled?

- ◯ Yes
- ◯ No

Do you feel that the quality of your care has been decreased?

Not at all — Somewhat — Very much

(Place a mark on the scale above)

Are you concerned that you or your baby are not receiving the care that you need?

Not at all — Somewhat — Very much

(Place a mark on the scale above)

Are you able to bring your partner or support person to your appointments?

- ◯ Yes
- ◯ No

Do you have any other comments regarding the rules for the involvement of your partner or your support person in your antenatal care?	______________________________
Have you made any changes to your birth plan because of the COVID-19 pandemic?	◯ Yes<br>◯ No
Which changes have you made to your birth plan (check all that apply):	☐ Birth location<br>☐ Support people<br>☐ Childcare arrangements<br>☐ Other
Please specify	______________________________
Do you currently have (or have you had) trouble accessing other health services during the COVID-19 pandemic? (e.g., massage, physiotherapy, etc.)	◯ Yes<br>◯ No
Which health services are (were) hard to access for you (check all that apply):	☐ Massage<br>☐ Chiropractic<br>☐ Physiotherapy<br>☐ Acupuncture<br>☐ Psychological Counselling<br>☐ Other
Please specify	______________________________
During the COVID-19 pandemic, I have felt more alone than usual.	Not at all — Somewhat — Very much so<br>*(Place a mark on the scale above)*
How has the COVID-19 pandemic affected your relationship with your circle of friends and family outside of your household?	It has strained our relationship — Not much has changed — It has brought us closer together<br>*(Place a mark on the scale above)*
If you would like to tell us more about any of your answers in the above section(s), please do so here:	______________________________

Mental wellbeing is an important part of overall health. We are interested in how women who are pregnant during the COVID-19 pandemic are doing. Below are questions about what you have been feeling, thinking, and doing.

How would you rate your distress levels at the peak of the pandemic in the UK?

- ◯ 10 (Extreme distress)
- ◯ 9
- ◯ 8
- ◯ 7
- ◯ 6
- ◯ 5 (Moderate distress)
- ◯ 4
- ◯ 3
- ◯ 2
- ◯ 1
- ◯ 0 (No distress)

Please choose the number that best describes how much distress you have been experiencing in the PAST 7 DAYS including today.

- ◯ 10 (Extreme distress)
- ◯ 9
- ◯ 8
- ◯ 7
- ◯ 6
- ◯ 5 (Moderate distress)
- ◯ 4
- ◯ 3
- ◯ 2
- ◯ 1
- ◯ 0 (No distress)
