# Supplementary material for: Prenatal maternal mental health and resilience in the United Kingdom during the SARS-CoV-2 pandemic: a cross- national comparison
Source: Front Psychiatry. 2024 Sep 26;15:1411761. doi: 10.3389/fpsyt.2024.1411761 (PMC11466367; doi:10.3389/fpsyt.2024.1411761)
Supplement: Supplementary file 2 [file Image2.pdf]

Supplementary Figure 2:

A: Receiver operating characteristic curve for the logistic regression model of EPDS in association with resilience factors.

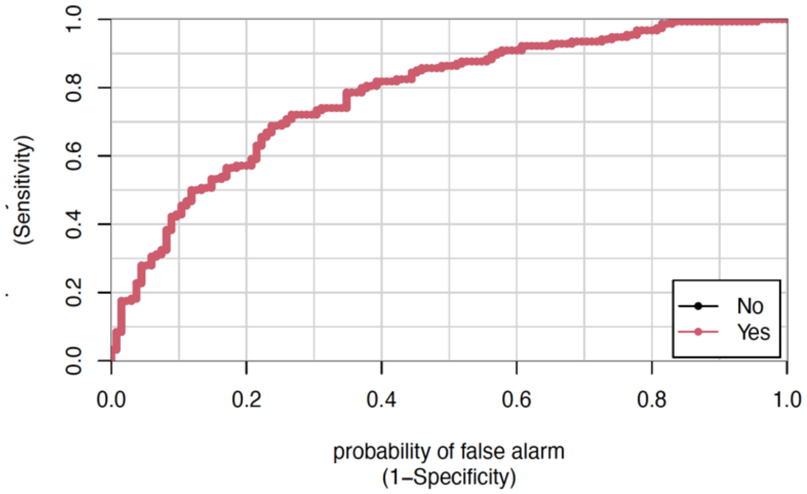

For a 0.5 probability threshold:

Accuracy: 0.7128
Sensitivity: 0.6815
Specificity: 0.7403
Kappa: 0.4223

B: Receiver operating characteristic curve for the logistic regression model of PROMIS anxiety in association with resilience factors.

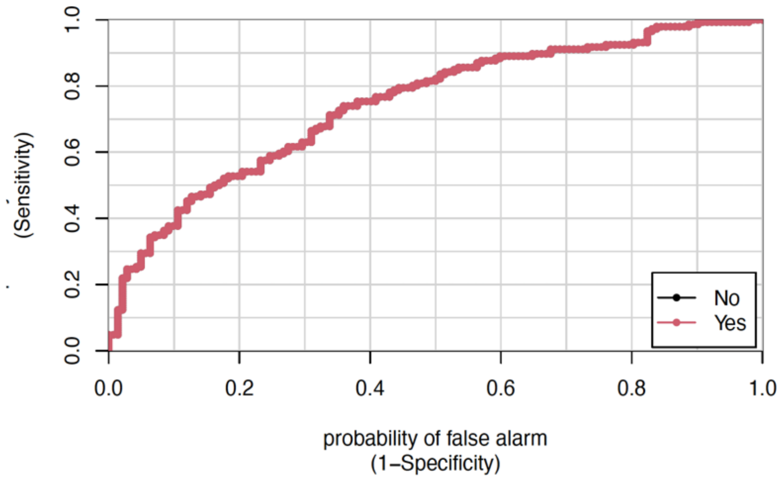

For a 0.5 probability threshold:
Accuracy: 0.6736
Sensitivity: 0.6761
Specificity: 0.6712
Kappa: 0.3472

C: Receiver operating characteristic curve for the logistic regression model of PROMIS anger in association with resilience factors.

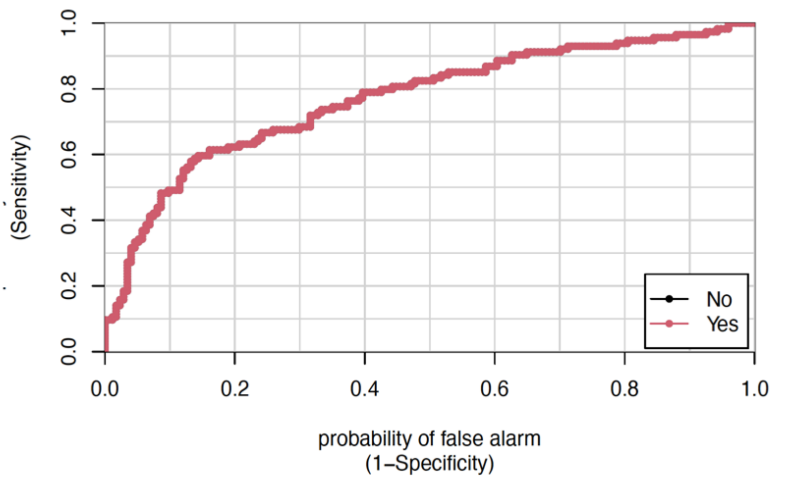

For a 0.5 probability threshold:

Accuracy: 0.7535
Sensitivity: 0.8563
Specificity: 0.5965
Kappa: 0.4677
