# Supplementary material for: Prenatal maternal mental health and resilience in the United Kingdom during the SARS-CoV-2 pandemic: a cross- national comparison
Source: Front Psychiatry. 2024 Sep 26;15:1411761. doi: 10.3389/fpsyt.2024.1411761 (PMC11466367; doi:10.3389/fpsyt.2024.1411761)
Supplement: Supplementary file 3 [file Table1.docx]

**Supplementary Table 1A:** EPPOCH study participant demographics – United Kingdom

| ***Family composition*** |  |
| --- | --- |
| No children | 15.92% |
| One other child | 54.75% |
| Two other children | 17.89% |
| Three or more other children | 11.44% |
| ***Residence in the UK*** |  |
| England | 81.10% |
| Scotland | 9.24% |
| Wales | 6.18% |
| Northern Ireland | 3.48% |
| ***Self-identification of ethnic/racial background****** | |
| Arabic | 0.06% |
| Black/African/Caribbean/Black British | 0.94% |
| Chinese | 0.18% |
| English/Welsh/Scottish/Northern Irish/Irish | 89.58% |
| Gypsy/Irish traveler | 0.09% |
| Indian/Pakistani/Bangladeshi | 0.88% |
| Other Asian background | 0.49% |
| Other Caucasian background | 6.14% |
| Other non-Caucasian ethnic group | 1.64% |
| ***Education level*** |  |
| Bachelor's degree | 35.98% |
| Trade/technical/vocational school or business/community college | 29.81% |
| Graduate degrees: Masters, PhD, MD, JD, DDS | 17.57% |
| High school diploma | 15.15% |
| No high school diploma | 1.49% |
| ***Pre-existing medical conditions*** |  |
| Yes | 77.38% |
| -Asthma | 17.08% |
| -Irritable Bowel Syndrome | 12.86% |
| -PCOS | 9.48% |
| -Obesity | 8.4% |
| -Infertility | 4.52% |
| -Hypothyroidism | 3.17% |
| -Autoimmune disease | 1.72% |
| -Celiac disease | 1.14% |
| -Primary (chronic) hypertension | 0.98% |
| -Epilepsy | 0.95% |
| Inflammatory Bowel Disease (Crohn’s disease) | 0.80% |
| Hyperthyroidism | 0.71% |
| Inflammatory Bowel Disease (ulcerative colitis) | 0.55% |
| Heart disease | 0.52% |
| Kidney disease | 0.40% |
| Type 2 diabetes | 0.40% |
| Type 1 diabetes | 0.31% |
| None | 22.62% |

**Supplementary Table 1B:** PdP study participant demographics - Canada

| Measure | N | % | Mean | Standard deviation | Range |
| --- | --- | --- | --- | --- | --- |
| **Demographics** | | | | | |
| Age (years) | 1900 |  | 32.4 | 4.2 | 18.6–47.6 |
| -Family composition | | | | | |
| No children |  | 49% |  |  |  |
| One other child |  | 37% |  |  |  |
| Two other children |  | 11% |  |  |  |
| Three or more other children |  | 3.5% |  |  |  |
| -Ethnic background******* | | | | | |
| Caucasian |  | 87.1% |  |  |  |
| First Nations |  | 0.7% |  |  |  |
| Metis |  | 1.2% |  |  |  |
| Inuit |  | 0.1% |  |  |  |
| Black |  | 0.7% |  |  |  |
| Chinese |  | 1.6% |  |  |  |
| Filipino |  | 0.9% |  |  |  |
| Korean |  | 0.2% |  |  |  |
| West Asian |  | 0.4% |  |  |  |
| South Asian |  | 2.6% |  |  |  |
| Southeast Asian |  | 0.3% |  |  |  |
| Hispanic/Latinx |  | 1.1% |  |  |  |
| Mixed Race/ Other |  | 3.3% |  |  |  |
| **Mental health measures** | | | | | |
| Depression (EPDS) | 1764 |  | 10.7 | 5.3 | 0-30 |
| PROMIS anxiety T-score | 1757 |  | 60.1 | 8.1 | 36–83 |
| Pregnancy-related anxiety (PRAQ) | 1757 |  | 21.1 | 5.1 | 10-40 |
| PROMIS anger T-score | 1643 |  | 54.52 | 8.5 | 32.9-82.9 |
| **Support** | | | | | |
| Physical activity (GLTEQ) | 1947 |  | 33.1 | 21.2 | 0-119 |
| Interpersonal Support Evaluation List (ISEL) | 1674 |  | 40.1 | 6.3 | 12-48 |
| Social Support Effectiveness Questionnaire (SSEQ) | 1685 |  | 55.8 | 14.9 | 4–80 |
| Personal resilience (CD-RISC 2) | 1630 |  | 5.8 | 1.3 | 0-8 |

*Participants chose their ethnic/racial background from a list of predefined categories. This data was collected to demonstrate diversity in the study population and to ensure the generalizability of the findings.
